# Asymmetric Segregation of Damaged Cellular Components in Spatially Structured Multicellular Organisms

**DOI:** 10.1371/journal.pone.0087917

**Published:** 2014-02-13

**Authors:** Charlotte Strandkvist, Jeppe Juul, Kristian Moss Bendtsen

**Affiliations:** 1 University College London, CoMPLEX, London, United Kingdom; 2 University of Copenhagen, Niels Bohr Institute, Copenhagen, Denmark; Institut de Génétique et Développement de Rennes, France

## Abstract

The asymmetric distribution of damaged cellular components has been observed in species ranging from fission yeast to humans. To study the potential advantages of damage segregation, we have developed a mathematical model describing ageing mammalian tissue, that is, a multicellular system of somatic cells that do not rejuvenate at cell division. To illustrate the applicability of the model, we specifically consider damage incurred by mutations to mitochondrial DNA, which are thought to be implicated in the mammalian ageing process. We show analytically that the asymmetric distribution of damaged cellular components reduces the overall damage level and increases the longevity of the cell population. Motivated by the experimental reports of damage segregation in human embryonic stem cells, dividing symmetrically with respect to cell-fate, we extend the model to consider spatially structured systems of cells. Imposing spatial structure reduces, but does not eliminate, the advantage of asymmetric division over symmetric division. The results suggest that damage partitioning could be a common strategy for reducing the accumulation of damage in a wider range of cell types than previously thought.

## Introduction

Despite the numerous repair and degradation processes involved in cell maintenance, over time cells accumulate damaged and deteriorated material [1]–[3]. This accumulation of damage has been linked to the decline in replicative function and increased risk of apoptosis associated with cellular ageing[4]–[9].

In some unicellular organisms, damaged cellular components are segregated preferentially to one daughter cell during division and this asymmetry is thought to have evolved as a strategy for ensuring the longevity of the lineage [10], [11]. In the budding yeast *Saccharomyces cerevisiae*, such differential inheritance has been documented for senescence factors including extra-chromosomal rDNA circles [9], oxidatively damaged proteins [6], [10], and defective mitochondria [8], [12]. When followed over successive generations, the division and growth rate of the mother cell receiving the damaged components is seen to decline [13], [14], whereas the daughter cell displays full replicative potential.

Differential inheritance of damaged proteins has been documented for epithelial stem cells in the intestine, which divide asymmetrically to produce a stem cell and a differentiated cell [15]. With the exception of germ-line cells and differentiating stem cells, mitosis in mammalian cells is commonly thought to occur symmetrically with the equal distribution of cellular components between daughter cells [2]. However, human embryonic stem cells undergoing self-renewing divisions have been found to segregate irreversibly damaged proteins preferentially to one daughter cell [16]. In the study by Fuentealba and colleagues (2008), cell-fate determinants were distributed equally between daughter cells, but proteins targeted for degradation were segregated asymmetrically. Likewise, several other species assumed to reproduce by morphologically symmetric division have been found to distribute damaged proteins asymmetrically [14], [17].

The experimental evidence suggests that asymmetric partitioning of damage is a more common strategy than previously thought. Several mathematical models have been developed to study the role of damage segregation in unicellular organisms [11], [18]–[21]. However, the effect of damage segregation on the ageing process of mammalian tissue - i.e. a multicellular system of somatic cells that do not rejuvenate at cell division - has not previously been explored. Furthermore, the existing computational models of damage segregation assume that the spatial organization of cells does not significantly impact the population dynamics. This may be a valid assumption for yeast, however, stem cells tend to inhabit microenvironments - known as stem cell niches - with a high degree of spatial structure that is instrumental in regulating stem cell fate [22].

We therefore developed a mathematical model to study damage segregation in cell populations with and without spatial structure. In the model, each daughter cell receives an amount of damage at least equal to the pre-divisional cell. This makes the model applicable to the description of cellular components where damage is duplicated during the cell cycle. In most cells, intracellular degradation processes are active and consequently the amount of damage accumulates more slowly than in our model. We consider the limiting case where damage is irreversible.

To illustrate the applicability of the model, we consider the specific case of damage incurred by mutations to mitochondrial DNA (mtDNA). The accumulation of defective mitochondria in ageing tissue is thought to be a major contributor to cellular ageing and has been linked to a variety of degenerative diseases [1]. Differential inheritance of defective mitochondria has been documented in budding yeast [3], [5], [8], [12], but an active process of mitochondrial selection may also occur in mouse ovarian germ cells [23], [24]. The model presented here demonstrates the potential benefits of such segregation.

## Methods

To study the accumulation of damage and the associated decline in the longevity of a population of cells, we developed a mathematical model of 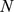 cells undergoing division and apoptosis, within a tissue that is homeostatic with respect to the number of cells. The damage level of a cell is denoted 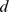 and represents the accumulation of mitochondrial DNA mutations; 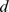 differs between cells and changes over time as each cell acquires additional damage. In the model, the probability of a cell going apoptotic is, in each time step, proportional to its damage level 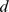. The apoptotic cell is removed from the tissue and immediately afterwards another cell divides to replace it. This mechanism ensures tissue homeostasis. The damage level of the dividing cell is denoted 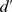 to distinguish it from that of the apoptotic cell.

Each cell contains multiple copies of mtDNA packaged into protein aggregates called nucleoids, with an average of 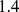 mtDNA molecules per nucleoid [25]. During the cell cycle, the number of nucleoids and mtDNA copies doubles. The mitochondrial genome is replicated and deletion-mutations and point-mutations are duplicated [26], [27]. It has been shown that nucleoids, in general, do not exchange genomic material [25], [28]. In the model, we consider the limiting case of irreversible damage from mutations to mitochondrial DNA. Consequently, each daughter cell, as a minimum, inherits the damage level 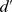 of the parent cell.

Between cell divisions, the mitochondrial genome may acquire additional damage 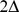, this occurs with a probability 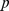. The mechanisms for segregation of mitochondrial DNA at cell division are not fully understood [29]. Rather than attempting to model the segregation mechanism, we consider the limiting cases of completely symmetric or completely asymmetric segregation of additional damage. No cells are allowed a damage level that exceeds 1.

**Figure 1:**
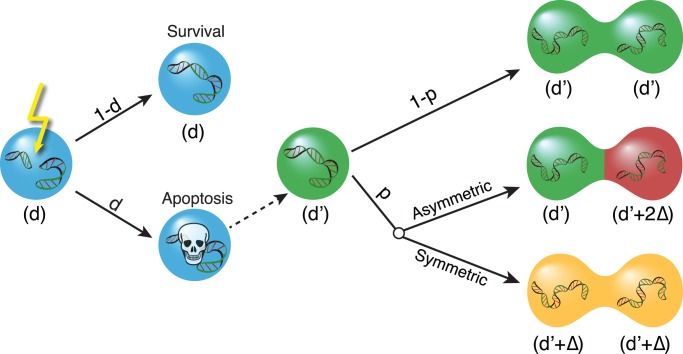
Schematic presentation of one time step of the computational model. Different colors correspond to different damage levels. In each time step of the computational model, a cell is selected at random. The cell has a probability of going apoptotic, given by its damage level 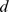, in which case another random cell, with damage level 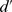, proliferates to replace it. If the selected cell does not go apoptotic, it remains in the cell population unchanged. The probability that the mitochondrial genome has acquired additional damage 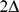 since the last cell division is 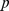. The daughter cells therefore have a probability 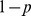 of inheriting a damage level of 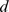 and a probability 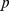 of inheriting additional damage. This may be distributed symmetrically, with both cells receiving 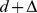 damage, or it may be segregated asymmetrically, leading to one cell with a damage level of 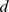 and one with a damage level of 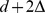. We define one time unit of the simulation to be 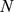 time steps, such that, on average, each cell is selected once per time unit.

Thus, the three parameters of the model are (i) the initial damage level 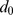 of all cells, (ii) the probability 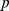 of acquiring additional mutations between cell divisions, and (iii) the additional damage accumulated during the cell cycle 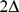. We will also refer to 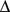 as the fragility of the system, in accordance with previous work [30]. The dynamics of the model are shown schematically in figure 1.

In addition to the well-mixed system, we also consider the effect of spatial structure. This is done by considering a system of cells arranged on a one dimensional line, with periodic boundary conditions, and imposing the constraint that cells undergoing apoptosis can only be replaced by proliferating neighbouring cells. Mathematically, a system of cells arranged spatially in two or three dimensions falls in between the one-dimensional and the well-mixed (mean field) case. Consequently, results that hold for both the one-dimensional and the well-mixed system can be expected to generalize to the case of two and three dimensions.

The mathematical model was written in MATLAB and C++, and the code is available upon request. Simulations were carried out for different parameter values, system sizes, with symmetrical or asymmetrical replication, and with and without spatial structure.

## Results

**Figure 2:**
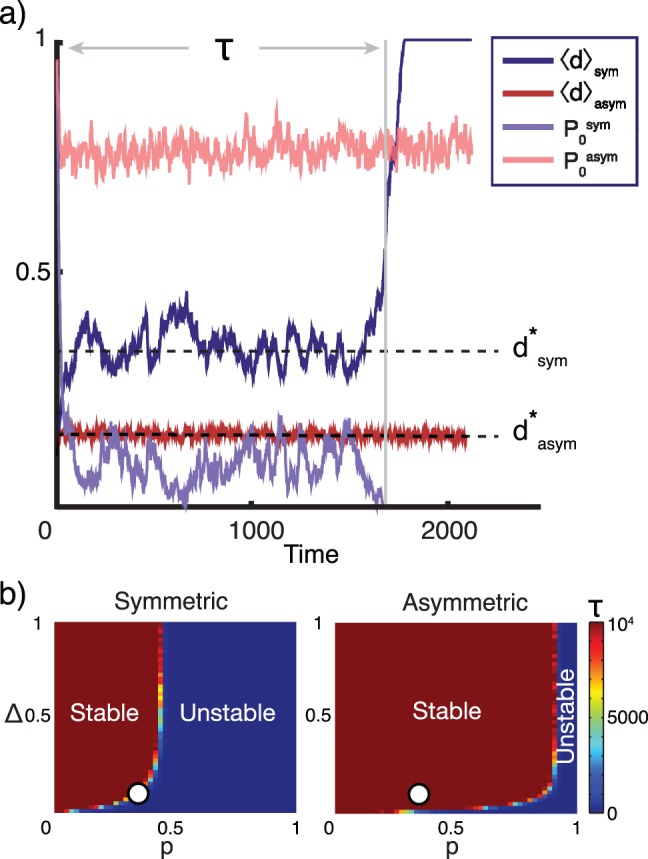
a) Damage level development. Cell populations that divide asymmetrically have a smaller average damage level 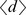 and a higher fraction 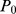 of cells with the initial damage level, compared to cells that divide symmetrically. When the number of undamaged cells fluctuates down to zero, the average damage level will quickly increase from the steady state value 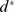 to 1, corresponding to a system collapse. This happens after a characteristic time 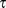. Parameters used: 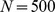, 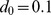, 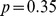, 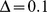. Other choices of parameters yield similar results. **b) Stability of steady state.** The time 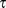 before system collapse decreases with the mutation probability 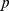 and increases with higher mitochondrial fragility 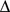. Notably, cell populations that divide asymmetrically stay in steady state for much longer time than populations with symmetric division. White circles represent the set of parameters used in panel a.

As the simulation progresses, cells gradually acquire mtDNA mutations and the average damage level of the system 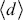 increases from the initial value of 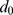. Since mutations are irreversible, the steady state where 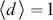 is absorbing. However, as seen in figure 2a, an additional transient steady state exists, characterized by a damage level 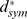 for symmetric divisions and 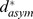 for asymmetric division. The steady state is sustained by cells with high levels of damage more frequently going apoptotic and being replaced by cells with fewer mtDNA mutations. As seen in figure 2a, the steady state damage level is higher when damage is distributed symmetrically upon cell divisions than when damage is segregated.

The steady state is only temporarily stable; eventually the fraction of cells, 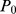, with the initial damage level, fluctuates down to zero and the system collapses to the state 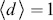. Biologically, this corresponds to the cell population having finite longevity. The characteristic time for this process is denoted by 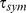 and 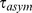 for the symmetrically and asymmetrically dividing system, respectively. Comparing the dependence of 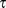 on the parameters 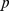 and 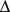 in figure 2b in the symmetric and asymmetric case, it is evident that damage segregation dramatically increases the longevity of the cell population. This is in agreement with the results of Erjavec and co-workers [18]. As expected, a decrease in the mutation probability 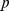 or the initial damage level 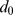 also increases the characteristic time for the collapse of the system, provided that 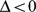.

Somewhat counterintuitively, increasing the fragility 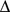 leads to a more stable system, as may also be seen from figure 2b. As reported elsewhere [30], if the acquired damage is associated with a high likelihood of apoptosis, defective cells are more effectively replaced by less damaged cells, thereby reducing the overall damage level of the system.

**Figure 3:**
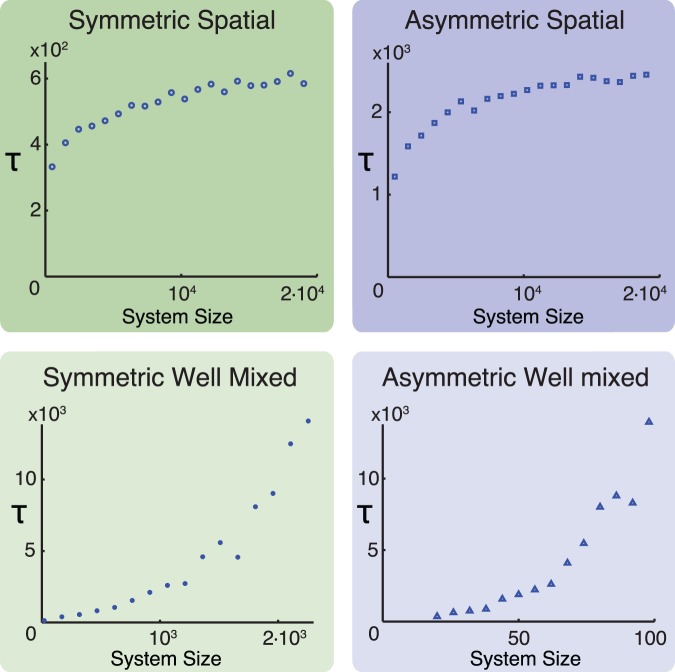
Effect of spatial structure and damage segregation. For well mixed systems, where apoptotic cells can be replaced by any other dividing cell in the population, the time 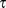 before system collapse increases drastically with the number of cells in the population. In systems with spatial structure, where apoptotic cells can only be replaced by neighboring cells, 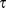 saturates with increasing 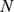, corresponding to an €effective system size for each cell. Both for spatial and well mixed systems, asymmetric division significantly increases the longevity of the cell population compared to symmetrically dividing populations of the same size. Notice that the asymmetric well mixed system has been studied using smaller cell populations due to the more rapid divergence of 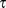 relative to the symmetric well mixed system. Each data point represents the median time before collapse out of 20 simulations. Parameters used: 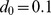, 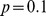, 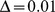. Other choices of parameters yield similar results.

In a well-mixed system, where an apoptotic cell can be replaced by any proliferating cell, the characteristic time for the transient steady state increases drastically with system size, as seen in figure 3. The figure also shows the results for a one-dimensional spatially structured system of cells with either symmetric or asymmetric distribution of damage. In both cases, a transient steady state exists for same parameters 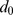, 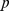, and 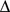. As in the well-mixed system, damage segregation increases the longevity of the cell population. However, imposing spatial structure diminishes the effect of system size and reduces the stability of the system since there is a tendency for defective cells to be replaced by other defective cells.

**Figure 4:**
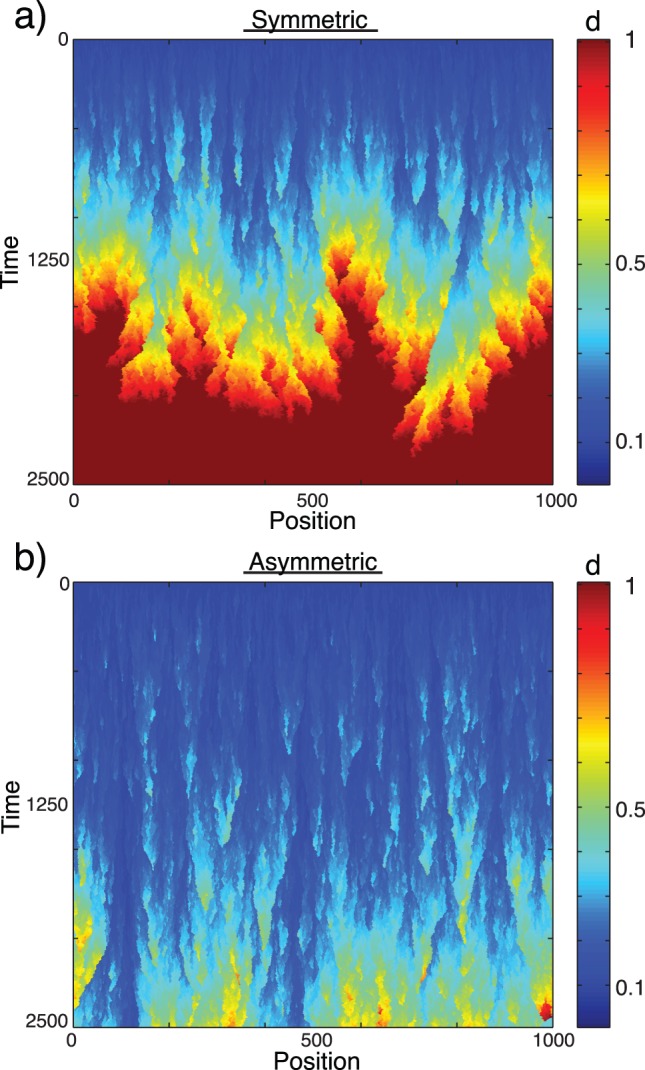
Time development of a spatially organized cell population that divides symmetrically (a) and asymmetrically (b). Initially, all cells have damage level 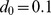, but soon clusters of damaged cells occur. The boundary between damaged and less damaged clusters performs a random walk with a drift towards the more damaged cells, since these are less likely to go apoptotic. Eventually the system will collapse to a state where all cells have damage level 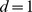. This happens much sooner for a symmetrically dividing cell population than for one that divides asymmetrically. The parameters are: 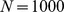, 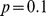, 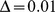. Other choices of parameters yield similar results.

Figure 4a and 4b show how the damage level of the system develops over time for a spatially structured system where damage is distributed symmetrically and asymmetrically, respectively. Cells that go apoptotic can only be replaced by neighbouring cells, which locally leads to strong correlations in damage levels and to the propagation of mutant mtDNA in the population. In both plots, clusters of defective cells develop and expand in space, but it is more pronounced in the system without damage partitioning. The boundary between clusters with many mutations and clusters with few, will perform a random walk with a drift towards the defective group, since these are more likely to be removed by apoptosis.

As the simulations demonstrate, the system displays a transient steady state for both symmetric and asymmetric distribution of damage. For a well-mixed system in this regime, the mathematical model presented above is analytically tractable.

In a system proliferating by symmetric division, a cell with 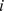 mutations is assigned a damage level of 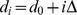. The fraction of such cells in the system is denoted 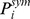. This fraction decreases if a cell goes apoptotic (which occurs at a rate of 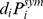) or if it acquires additional mutations before dividing to replace an apoptotic cell (rate 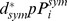). The fraction increases if the cell does not mutate prior to cell division (rate 
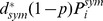
). If a cell with 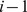 mutations acquires an additional mutation before dividing, two new cells, each with 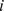 mutations, are generated. This occurs at a rate of 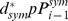 and, consequently, 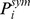 increases at twice this rate. The equations governing the system are
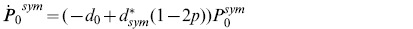
(1)




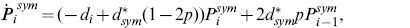
(2)Here the dot represents differentiation with respect to time.

In steady state the left hand side vanishes, turning (1) into an expression for the steady state damage level 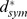, which may be inserted into (2) to yield a recurrence relation for the steady state.
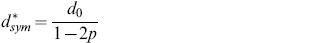
(3)




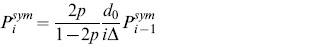
(4)


The normalized solution to 4 is a Poisson distribution with mean 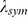.
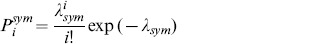
(5)




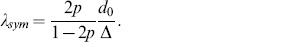
(6)


From (3) it is evident that, for 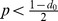, the steady state value 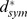 only takes values between 0 and 1. This is in agreement with the observations in figure 2b.

In a system where damage is distributed asymmetrically, one daughter cell receives all mitochondrial DNA mutations the mother cell has accumulated since last division, and as a result the damage level of the cell increases by 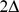. The probability of having a cell in the system that has received the mutational increment in 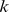 of the cell divisions it has undergone is given by 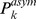. Following the same procedure as above, we obtain the governing equations.
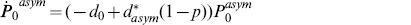
(7)




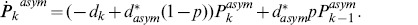
(8)


As before, demanding that the time derivatives vanishes, yields a steady state damage level of 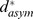 and a Poisson distribution for the mean number of mutational increments per cell 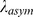:
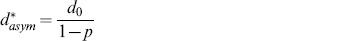
(9)




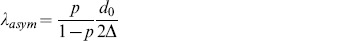
(10)




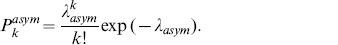
(11)


From the analytical treatment of the model, the damage levels at steady state are 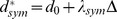 and 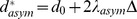. As seen in figure 2a, these values are in agreement with the results of the computational simulation.

From (3) and (9) it is evident that 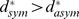 for all parameter values. This implies that asymmetric segregation of damage is a more effective mechanism for reducing the amount of mtDNA damage accumulated in the population, than symmetric division.

The mean number of cells with the initial damage level is given by 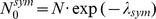 and 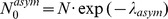. Since 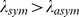, cell populations that partition damage have a larger proportion of unmutated cells at steady state and consequently 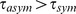 for the same set of parameters. Asymmetric segregation of damage thus increases the longevity of the population.

When the number of cells with the initial damage level fluctuates down to zero, a new steady state can be identified using the substitutions 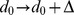 and 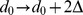 for the symmetric and the asymmetric system, respectively. However, this increases the mean number of mutations, making these new steady states less stable. This explains the rapid collapse of the system observed in figure 2a.

## Discussion

The mathematical model presented here demonstrates that the asymmetric distribution of damage reduces the overall damage level and increases the longevity of a population of cells in both spatially structured and unstructured systems. We have shown analytically that these results hold for all parameter values, implying that damage segregation, in this model, is always advantageous. This is in agreement with several computational studies published recently [11], [18]–[21].

In their model, Erjavec and colleagues (2008) assume that damaged proteins have no intrinsic toxicity but that their accumulation prolongs the time it takes for the cell to acquire the critical number of intact proteins required for cytokinesis. Given that condition, damage partitioning enhances the population fitness of a unicellular organism and allows the lineage to withstand higher levels of damage before system collapse for all damage rates analysed.

The work by Ackermann and colleagues (2007) demonstrates that the advantage of segregating damage depends on how the damage accumulated in a cell affects survival and reproduction. Their results indicate that asymmetry is selected for evolutionarily if asymmetric phenotypes have at least the same expected number of surviving progeny as symmetric phenotypes.

In the model presented here we have assumed that the probability of a cell going apoptotic is proportional to its damage level, which corresponds to the case of a linear survival function in [11] and our results are therefore in agreement. This form of the survival function has been validated experimentally in the bacterium Caulobacter crescentus [11]. Furthermore, even though Escherichia coli reproduces by morphologically symmetric division, studies have found that the cell inheriting old components exhibits a decreased reproductive output and an increased probability of apoptosis [14].

An important distinction between our work and that of [18] and [11] is that we consider cells that are not rejuvenated at cell division. Rather, each daughter cell receives an amount of damage at least equal to that of the predivisional cell. This makes the model applicable to defective mitochondrial DNA and other cellular components known to be duplicated during cell division. If damage is diluted at division - either by the repair of damage or the production of undamaged copies - a steady state similar to the one described here will occur when dilution balances the rate at which new damage is accumulated. In this case, the steady state will be permanent and the longevity of the cell population will no longer be finite.

The models presented in [11], [18]–[20] describe a population of unicellular organisms and the focus is therefore on how damage segregation affects the growth rate. Our model, on the other hand, considers somatic cells within a tissue and hence the population size is kept constant.

Mechanisms for actively generating damage asymmetry are well established in several organisms [31] and these mechanisms appear to be able to accommodate changes in environmental conditions that affect the damage load cells are exposed to. In budding yeast, increasing the levels of oxidatively damaged proteins increases the damage asymmetry between the mother and daughter cell [10]. Furthermore, a mutant strain of budding yeast, characterized by the inability to segregate functioning mitochondria preferentially to the daughter cell, suffered an extensive loss of viability resulting in clonal senescence [8]. This is consistent with the expectations of the model presented here.

As well as damage asymmetry, the process of cell division may in itself be important for stabilizing the population. In mammalian cells, defective mitochondria have been shown to accumulate to a greater extent in post-mitotic tissues, such as brain and muscle, than in dividing tissue [32]–[34].

Our investigation of the effect of spatial structure was motivated by experimental reports of the asymmetric distribution of damage in dividing stem cells [15], [16]. The result that segregation reduces the level of damage accumulated in spatially structured cell populations suggests that it could be a strategy for reducing stem cell ageing even for cells that divide symmetrically with respect to cell fate.

The model presented here extends the theoretical framework for investigating the potential benefits of damage partitioning to multicellular organisms and spatially structured systems. Our results indicate that the asymmetric distribution of damage - and in particular the segregation of defective mitochondria - is advantageous in both spatially structured and unstructured systems for all parameter values. Damage segregation might be a common strategy even for somatic cells that are not rejuvenated at cell division.
